# Surgery Experience with Calcified Amorphous Tumor of the Mitral Valve Complicated by Infective Endocarditis

**DOI:** 10.70352/scrj.cr.25-0682

**Published:** 2025-11-28

**Authors:** Kazuki Iwamoto, Koji Furukawa, Risa Meiri, Ayaka Iwasaki, Kousuke Mori, Shuhei Sakaguchi, Mayuko Uehara, Hirohito Isihi, Kazunari Maekawa, Atsushi Yamashita

**Affiliations:** 1 Division of Cardiovascular Surgery, Department of Surgery, Faculty of Medicine, University of Miyazaki, Miyazaki, Miyazaki, Japan; 2 Department of Pathology, Faculty of Medicine, University of Miyazaki, Miyazaki, Miyazaki, Japan

**Keywords:** calcified amorphous tumor, infective endocarditis, mitral valve, surgery

## Abstract

**INTRODUCTION:**

Cardiac calcified amorphous tumors (CAT) are non-neoplastic cardiac lesions composed of calcified nodules. These lesions are associated with underlying factors such as hypertension, diabetes mellitus, and end-stage renal dysfunction. However, its association with infective endocarditis remains unclear.

**CASE PRESENTATION:**

The patient was a 72-year-old woman with a medical history of diabetes mellitus, hypertension, and ossification of the posterior longitudinal ligament. However, no renal dysfunction was observed. For the past 2 months, she had been prescribed antibiotics for low-grade fever. The patient exhibited signs of cerebral and splenic infarctions, along with a vegetative mitral valve apparatus. Cardiac CT showed a posterior leaflet with a tumor-like appearance and calcification. Intraoperative findings revealed no calcification of the mitral annulus. However, the mitral valve leaflets were markedly thickened, with a vegetate attached to the posterior leaflet. The vegetation was then removed and the anterior leaflet was easily excised. The posterior leaflet, which was markedly thickened with calcification, was excised using a No. 15 scalpel and incised along its border with the left ventricular posterior wall. After irrigation, the left ventricular posterior wall and mitral annulus were covered with bovine pericardium and a 25-mm biological valve was implanted in the supra-annular position. The results of pathological and bacterial examinations were consistent with those of a case of CAT complicated by infective endocarditis. The patient’s postoperative course was uncomplicated.

**CONCLUSIONS:**

We encountered a rare case of CAT complicated by infective endocarditis. Complete resection of the CAT in combination with pericardial patch reconstruction of the left ventricular posterior wall, mitral annulus, and mitral valve replacement using a bioprosthesis yielded favorable results.

## Abbreviations

CAT
: calcified amorphous tumor
DM
: diabetes mellitus
ESRD
: end-stage renal disease
IE
: infective endocarditis
MAC
: mitral annular calcification
MV
: mitral valve
OPLL
: ossification of the posterior longitudinal ligament

## INTRODUCTION

Cardiac CATs are relatively uncommon nonneoplastic cardiac lesions characterized by the presence of calcified nodules.^1–3)^ Their etiology is multifactorial, including, but not limited to, hypertension, DM, and ESRD.^1–3)^ While often discovered incidentally, CATs can cause chest discomfort, other thoracic symptoms, or embolic symptoms, such as cerebral infarction, depending on their size and location. However, their association with IE is extremely rare,^4–6)^ and surgical experience with mitral valve CATs complicated by IE is limited to only 2 cases within the scope of our search.^5,6)^ Here, we present our experience with the surgical intervention for MV CAT complicated by IE.

## CASE PRESENTATION

A 72-year-old woman with a history of DM, hypertension, and OPLL of the thoracic spine was prescribed antibiotics for 2 months by her primary care physician for generalized fatigue and low-grade fever. However, her symptoms worsened, necessitating hospitalization at another facility. Although her blood cultures were negative, her C-reactive protein level was elevated at 16.5 mg/dL. MRI of her head revealed scattered subacute cerebral infarction lesions in both cerebral hemispheres. Contrast-enhanced CT scan showed splenic infarction. Transthoracic echocardiography revealed thickening of the posterior MV leaflet and a mobile structure, indicating IE of the MV. After antibiotic treatment, the patient was transferred to our hospital for surgery.

**Fig. 1:**
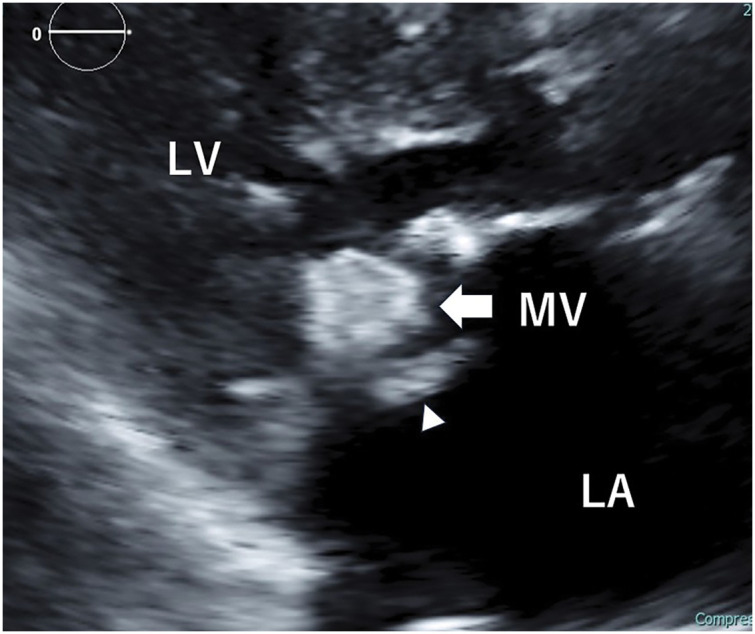
Parasternal long axis view of a transthoracic echocardiogram. The posterior mitral valve leaflet exhibits a calcified, tumor-like appearance (arrow) accompanied by mobile vegetation (arrowhead). LA, left atrium; LV, left ventricle; MV, mitral valve

**Fig. 2:**
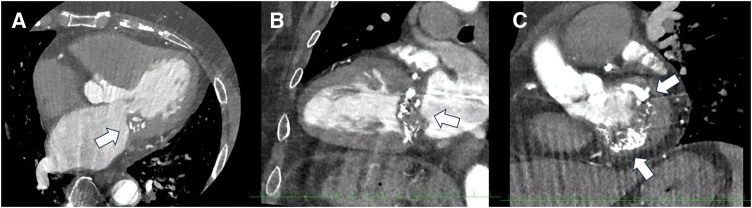
Cardiac CT. (**A** and **B**) The posterior leaflet of the mitral valve exhibits a tumor-like appearance with calcification (arrow). (**C**) A tumor-like appearance with calcification (arrow) was extending from the anterior commissure to the posterior commissure.

**Fig. 3:**
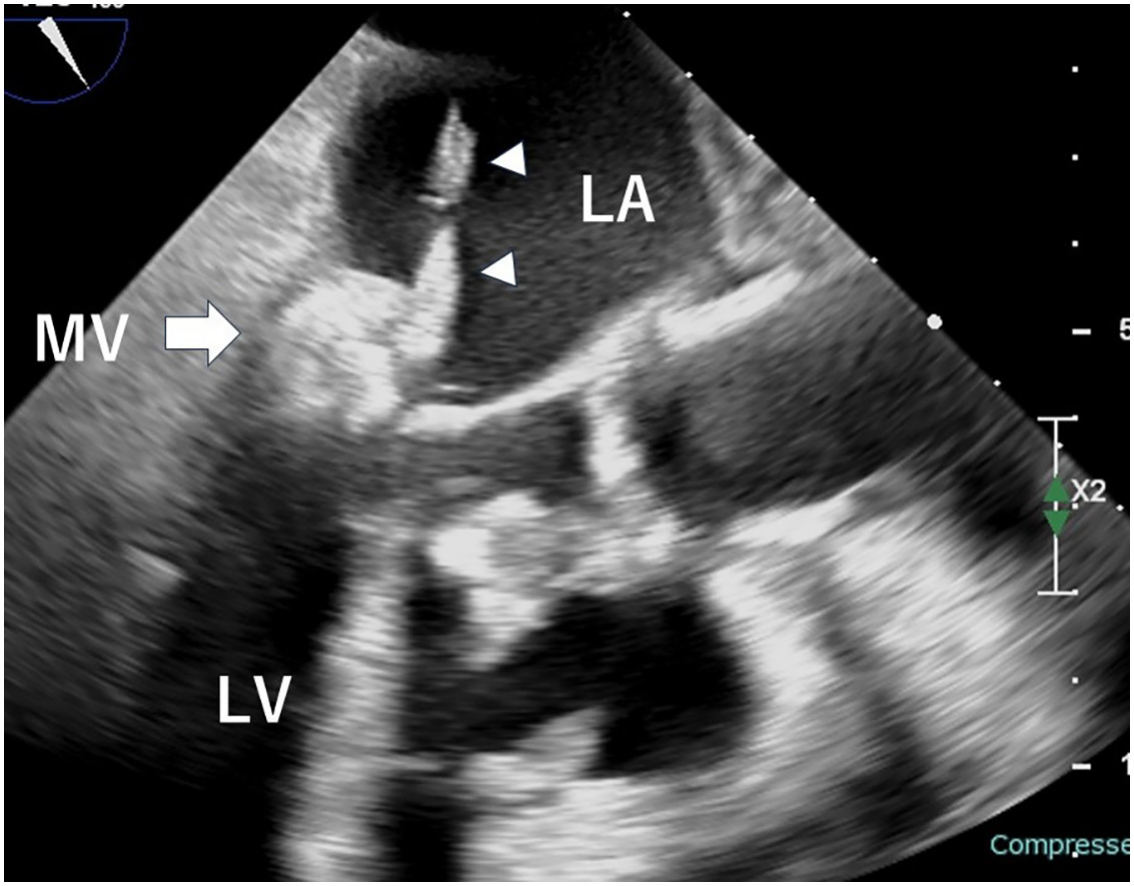
Transesophageal echocardiogram. A 3-cm long vegetation (arrowhead) was attached to the tumor-like posterior leaflet (arrow). LA, left atrium; LV, left ventricle; MV, mitral valve

The patient had no history of dental treatment prior to this episode. On transfer, the patient was alert and oriented. The patient had a blood pressure of 135/80 mmHg, a pulse rate of 92 beats per minute, and a body temperature of 36.9°C. A complete blood count revealed a white blood cell count of 5400/μL, a hemoglobin level of 13.9 g/dL, a hemoglobin A1c percentage of 6.5%, and a creatinine level of 0.48 mg/dL. The estimated glomerular filtration rate was 94 mL/min/1.73m^2^. Brain natriuretic peptide levels were recorded at 74 pg/mL. C-reactive protein levels measured 1.53 mg/dL and serum calcium concentration was 8.2 mg/dL (8.5–10.5 mg/dL). Additionally, documented phosphorus levels 3.2 mg/dL (2.7–4.6 mg/dL). Electrocardiography revealed sinus rhythm. Chest radiography revealed a cardiothoracic ratio of 53%. Echocardiography revealed a left ventricular ejection fraction of 70%, left ventricular end-systolic dimension of 21 mm, and left atrial dimension of 46 mm. The MV displayed a calcified tumor-like thickening of the posterior leaflet with mobile vegetation (**Fig. 1**). Trivial mitral regurgitation, mild mitral stenosis, and trivial tricuspid regurgitation were also observed. Cardiac CT showed a tumor-like mass in the posterior MV leaflet measuring 40 × 15 mm. The mass showed significant calcification extending from the anterior to the posterior commissure, suggesting CAT (**Fig. 2**). Preoperative transesophageal echocardiography revealed a 3-cm-long vegetation on the MV (**Fig. 3**).

**Fig. 4:**
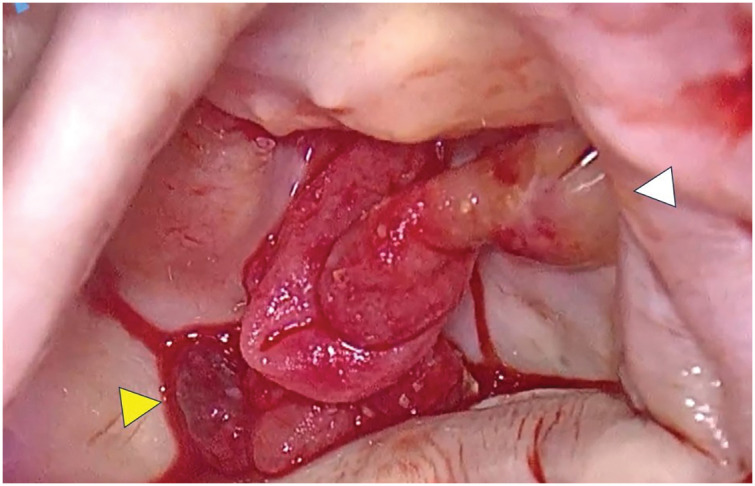
Intraoperative findings. The club-shaped vegetation was attached to the posterior commissure (white arrowhead), and the cauliflower-shaped vegetation was attached to P2 (yellow arrowhead).

**Fig. 5:**
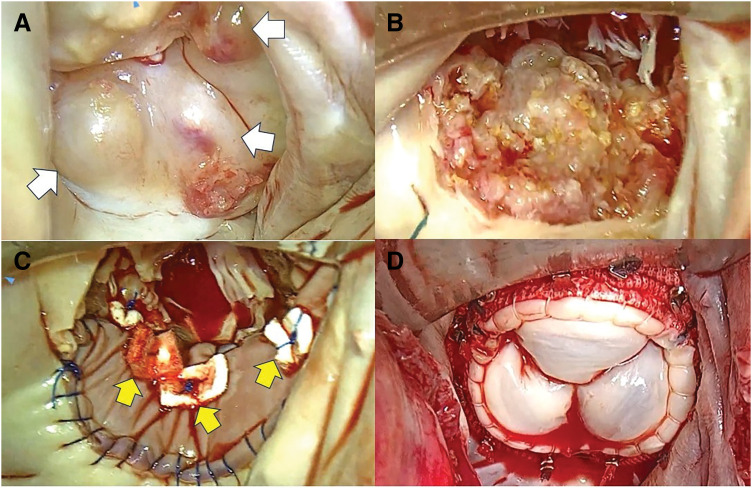
Intraoperative findings. (**A**) After the vegetations were removed, the mitral valve appeared grayish-white and bulging (white arrow). (**B**) Appearance of the left ventricular posterior wall and posterior mitral annulus after resection of a cardiac calcified amorphous tumor. (**C**) Appearance after patch reconstruction. In addition to the continuous sutures, several mattress sutures were added (yellow arrow). (**D**) Appearance after bioprosthetic valve replacement.

A superior transseptal approach was used to ensure an optimal field of view. The 3-cm club-shaped and cauliflower-shaped vegetations were attached to the posterior commissure and P2, respectively (**Fig. 4**). After eliminating vegetation, no MAC was identified within the posterior leaflet annulus. The MV leaflets were greyish white and bulging (**Fig. 5A**). Excision of the anterior leaflet was uncomplicated; however, removal of the posterior leaflet using scissors was more challenging. Consequently, the border of the left ventricular posterior wall was meticulously incised and dissected using a No. 15 scalpel (**Fig. 5B**). After irrigation, the left ventricular posterior wall scraping site and posterior mitral annulus were covered with a 7-cm-long elliptical piece of bovine pericardium (**Fig. 5C**). Because it was difficult to preserve the native chordae tendineae, an artificial chorda was used to restore continuity between the anterior papillary muscle and the MV annulus. Thereafter, a 25-mm epic bioprosthetic valve (SJM, St Paul, MN, USA) was placed in the supra-annular position (**Fig. 5D**). Weaning from the cardiopulmonary bypass was performed without difficulty. The aortic cross-clamp, cardiopulmonary bypass, and total operative times were 229, 277, and 470 min, respectively. A surgical video is available in the **Supplementary Video**.

**Fig. 6:**
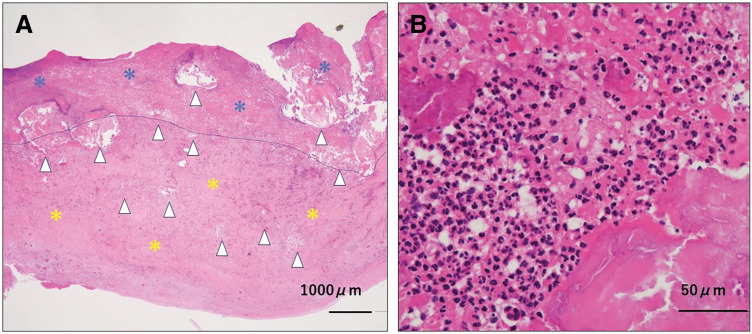
Pathological Findings. (**A**) Nodular calcified material depositions (arrowheads) are embedded in thrombus (blue asterisk) and granulation tissue (yellow asterisk), resulting in marked thickening of the leaflet. Dotted line indicates the interface between the thrombus and the inflamed leaflet. (**B**) Dense neutrophilic infiltrate is noted associated with infectious endocarditis juxtaposed with nodular calcification.

Postoperative echocardiography revealed a 60% left ventricular ejection fraction and no leakage around the MV prosthesis. Corynebacterium striatum was identified in the vegetation obtained during surgery. Pathology revealed a significantly enlarged posterior leaflet of the MV with a thrombus on the ventricular side. Numerous calcifications were observed within the thrombus and the MV granulation tissue (**Fig. 6A**). Furthermore, extensive neutrophilic infiltration was observed (**Fig. 6B**), consistent with cases of CAT complicated by IE. The patient was administered intravenous vancomycin for 30 days based on susceptibility testing. On POD 20, the patient was transferred and continued antibiotic therapy under the care of a referring physician. The patient was discharged on day 38 without any complications.

## DISCUSSION

CAT is a non-neoplastic cardiac lesion consisting of calcified nodules against anaplastic, chronic inflammatory, and amorphous fibrous tissues, and was first reported by Reynolds et al. in 1997.^1)^ It is frequently associated with hypertension, DM, and ESRD and has been reported to correlate with abnormalities in calcium and vitamin D metabolism, as well as coagulation disorders.^2,3)^ CAT can occur in any part of the heart; however, the MV is the most common site.^3)^ MV-CAT is strongly associated with MAC and ESRD.^2,3)^

Our patient had no MAC or ESRD and had normal serum calcium and phosphorus levels upon admission. However, the patient had a history of hypertension and DM as well as prior treatment for OPLL, which is also believed to be linked to calcium and vitamin D metabolism. These factors may have caused CAT. Furthermore, cases of CAT linked to IE are very uncommon, with only 3 documented cases reported thus far.^4–6)^ The role of infection as either a complication or cause of CAT remains uncertain. Kimura et al. reported a case of IE that activated the coagulation system and caused CAT formation and rapid growth.^6)^ Our patient had no previous transthoracic echocardiographic findings. However, the substantial pathological thrombus attached to the posterior leaflet and pervasive neutrophilic infiltration suggested that the CAT may have grown, as Kimura et al. proposed^6).^

Surgical procedures for CAT are based on the lesion location, size, and valvular dysfunction, ranging from tumor resection to valve repair or replacement.^7–9)^ In cases where the CAT infiltrates the left ventricular posterior wall or is complicated by the MAC, combined procedures may be necessary, including excision of both the CAT and MAC, pericardial patch reconstruction of the left ventricular posterior wall and mitral annulus, and MV replacement.^10)^

Surgery for MV CAT with IE has been reported in only 2 cases.^5,6)^ **Table 1** lists all the further details, including our case. All patients were women aged >70 years without ESRD. Large MV CATs require mitral annular reconstruction. Handa et al. reported 2 episodes of left ventricular rupture during surgery, highlighting the need for extreme caution in surgical intervention for giant infective MV CAT.^5)^

**Table 1:** Summary of surgical cases for infectious mitral valve calcified amorphous tumors

No	Author (Year)	Age	Sex	Past history	ESRD	Pathogenic bacteria	Size of CAT (mm)	MAC	Valvular disease	Surgery	Outcome
1	Handa et al.^5)^ (2021)	76	F	HT HL OP	No	Gemella Spp.	37 × 24	No	AR MR	AVR	LV rupture
										CAT resection	In-hospital death
										Annular reconstruction	
										MVR	
2	Kimura et al.^6)^ (2022)	82	F	HT	No	Not mentioned	14.9 × 12.1	Yes	Not mentioned	CAT resection	Discharge in good health
										MVR with bioprosthesis	
3	Our case	72	F	HT DM OPLL	No	Undetected	50 × 15	No	Mild MS	CAT resection	Discharge in good health
										Annular reconstruction	
										MVR	

AR, aortic regurgitation; AVR, aortic valve replacement; CAT, calcified amorphous tumor; DM, diabetes mellitus; ESRD, end-stage renal disease; F, female; HL, hyperlipidemia; HT, hypertension; LV, left ventricle; MAC, mitral annular calcification; MR, mitral regurgitation; MS, mitral stenosis; MVR, mitral valve replacement; OP, osteoporosis; OPLL, ossification of the posterior longitudinal ligament

In general, MV surgery requiring annular reconstruction with a pericardial patch requires a longer aortic cross-clamp time and raises concerns regarding the risk of atrioventricular groove dissociation.^11)^ However, when performed by a skilled team, the procedure can be performed relatively safely^11)^; favorable early and intermediate outcomes have been reported previously, even for annular abscesses.^12)^

In this case, contamination of the surgical field was minimized, and the appropriate layer was properly dissected by excising the CAT as a single mass as much as possible without fragmentation with a No. 15 scalpel.^13)^ To repair the posterior left ventricular wall and mitral annulus, we cut a bovine pericardial patch slightly larger than the debrided area and sutured it in place. In addition to the continuous sutures, we added several mattress sutures to prevent dehiscence of the suture lines (**Fig. 5C**).^11)^ To prevent periannular regurgitation and reduce the risk of left ventricular rupture, we selected an epic bioprosthetic valve with a rich sewing cuff and low struts, which yielded favorable results.^5)^ However, the postoperative follow-up period is short, and careful follow-up will be required going forward.

## CONCLUSIONS

We performed surgical intervention for giant MV CAT complicated by IE. Complete resection of the CAT was achieved, followed by reconstruction using a pericardial patch on the left ventricular posterior wall and mitral annulus and mitral valve replacement with a bovine bioprosthesis, yielding favorable outcomes.

## SUPPLEMENTARY MATERIALS

Supplementary Video
